# mRNA Expression of FGFR1 as Potential Marker for Predicting Prognosis of Surgical Resection of Small Cell Lung Cancer may be better than Protein Expression and Gene Amplification

**DOI:** 10.7150/jca.44476

**Published:** 2020-05-22

**Authors:** Jing Qin, Fajun Xie, Fenfang Wang, Hongyang Lu

**Affiliations:** 1 Graduate School, WenZhou Medical University, Wenzhou, 325035, P.R. China.; 2 Zhejiang Key Laboratory of Diagnosis and Treatment Technology on Thoracic Oncology (lung and esophagus), Institute of Cancer Research and Basic Medical Sciences of Chinese Academy of Sciences, Cancer Hospital of University of Chinese Academy of Sciences, Zhejiang Cancer Hospital, 310022, P.R. China.; 3 Department of Thoracic Medical Oncology, Institute of Cancer Research and Basic Medical Sciences of Chinese Academy of Sciences, Cancer Hospital of University of Chinese Academy of Sciences, Zhejiang Cancer Hospital, 310022, P.R. China.

**Keywords:** small cell lung cancer (SCLC), FGFR1, mRNA expression, amplification, protein expression, mutation, prognosis

## Abstract

**Purpose:** Fibroblast growth factor receptor 1 (FGFR1) alterations have been described in many cancers, including lung cancer, but the role has not been elucidated specifically in small cell lung cancer (SCLC). The present study aimed to identify the frequency of FGFR1 alterations among Chinese patients with surgically resected SCLC and the association with the clinicopathological characteristics and the survival were also investigated.

**Methods:** FGFR1 protein expression,* FGFR1* amplification, *FGFR1* mutations, and messenger RNA (mRNA) levels, were determined by immunohistochemistry (IHC), fluorescence *in situ* hybridization (FISH), polymerase chain reaction (PCR) and reverse transcription-polymerase chain reaction (RT-PCR), respectively in primary tumors from 33 patients with resected SCLC.

**Results:** 7/33(21.2%) of the specimens were positive for FGFR1 protein expression.* FGFR1* amplification was identified in 4/28 cases (14.3%). If the cut-off value was determined to be 3.5, FGFR1 mRNA positivity was considered in 7/33 cases (21.2%). However, no mutation was detected in the 33 SCLC postoperative tissue specimens. No significant association was observed between FGFR1 protein expression or amplification and clinicalcharacteristics or prognosis. There was a distinct trend for mRNA level and poor prognosis, including recurrence-free survival (RFS) (*p* = 0.07) and overall survival (OS) (*p*= 0.08), but they did not reach statistical significance.

**Conclusions**: As novel FGFR1-targeted therapies are developed, FISH, IHC, especially mRNA were detected, which should be considered as biomarkers of FGFR1 pathway dysregulation in SCLC.

## Introduction

Lung cancer is the most aggressive and lethal malignancies and ranks as the leading cause of cancer-related deaths worldwide including China 1,2. Small-cell lung cancer (SCLC) is exceptionally aggressive and constitutes approximately 15% of all lung cancers with different clinical and pathological features to non-small-cell lung cancer (NSCLC); thus, it is mostly diagnosed at late stages with systemic metastases. Although chemoradiotherapies are initially effective in patients with SCLC, responses are typically transient and recurrences arise rapidly in the vast majority of cases with poor survival outcome. Therefore, the median survival time of limited-disease and extensive-disease SCLC is 15-20 months and 8-10 months, respectively 3,4. Therefore, finding the potential novel targets and effective agents to improve the prognosis of SCLC patients is an urgent requisite.

Recently, large-scale genomic detections using next-generation sequencing (NGS) have been performed to elucidate the genomic profiles of SCLC. Although deletion or inactivation of *TP53* and *RB1* and amplifications of *MYC* family have been identified 5,6, effective targeted drugs for SCLC are yet lacking. Nevertheless, minority SCLC patients also harbor fibroblast growth factor receptor1 (FGFR1) amplification, generating a great interest in evaluating the role of FGFR1 as a driver oncogene and a promising therapeutic target. Preclinical evidence suggested that SCLC patients might benefit from FGFR inhibitor therapy 7,8. The FGFR1 inhibitor therapy is currently under clinical trials. However, the response rates did not reach anticipation, suggesting that the biomarkers used for enrolling into the FGFR tyrosine kinase inhibitor (TKI) trials were inaccurate. Previous clinical trials have screened patients with* FGFR* amplification and protein over-expression 7,9. However, basic research showed that FGFR1 mRNA and protein expression, not gene copy number, predict FGFR TKI sensitivity across all histopathological lung cancer 10. The latest study from a phaseⅠclinical trial suggested that rogaratinib, a novel kinase inhibitor of FGFR1-4, resulted in an encouraging antitumor activity, if screened by FGFR mRNA overexpressing cancers 11. In addition, the mutation in FGFR1 V561M gatekeeper drives the FGFR TKI AZD4547 resistance *in vitro* 12. However, very few or none studies examined FGFR1 mRNA expression and mutation with tissue samples from patients and assessed their values.

Thus, the present study aimed to comprehensively assesse the frequency of *FGFR1* gene amplification, protein expression, gene mutation and mRNA levels from a series of surgically resected primary SCLCs and investigated the correlation between their expressions and prognosis.

## Materials and methods

### Patient Population and Tumor Specimens

Formalin-fixed paraffin-embedded tumor samples were obtained from a unique series of 33 patients with SCLC, who underwent pulmonary resection between April 2008 and June 2014 at Zhejiang Cancer Hospital (Hangzhou, China) 13. Three patients underwent pneumonectomy with lymph node dissection, one patient received wedge resection with lymph node dissection, and 29 patients received lobectomy with lymph node dissection. All patients were diagnosed with conventional SCLC, and the pathological diagnosis was based on the standard criteria defined by WHO classification 14.

Specimens from 33 patients were subjected to immunohistochemistry (IHC), polymerase chain reaction (PCR), reverse transcription-polymerase chain reaction (RT-PCR) and medical records were reviewed to obtain clinical characteristics, including gender, age, smoking status, tumor stage, referring to our previous published research 13. Furthermore 28/33 were subjected to fluorescence in-*situ* hybridization (FISH) analysis, and medical records were reviewed to assimilate the clinical characteristics, including gender, age, smoking status, tumor stage (**Table 1**). The tumor stage was classified according to eighth edition of the TNM classification for lung cancer as follows: IA, 9 cases; IB, 1 case; IIA, none; IIB, 5 cases; IIIA, 12 cases; and IIIB, 1 case. The 28 specimens were obtained from 6 female and 22 male patients, aged 38-77 (median age, 58) years. The cohort comprised of 6 non‑smokers, 2 light smokers (≤10 pack-years), 2 moderate smokers (10-20 pack-years), and 18 heavy smokers (≥20 pack-years). The median pack-years of smoking history were 30. The present study was approved by the Ethics Committee of Zhejiang Cancer Hospital. As the patient specimens were collected in a retrospective approach, and the numbers of patients were deceased, exempt written informed consents were also approved by the Ethics Committee of Zhejiang Cancer Hospital. Finally, a total of 21 patients signed the written informed consent prior to surgery to preserve their specimens in the Biological Sample Bank of Zhejiang Cancer Hospital to be used for research.

### IHC of FGFR1 expression

IHC for FGFR1 was performed using FGFR1 antibody (Cat, #BA0485, Boster Biological Technology Co. Ltd). Briefly, after deparaffinization and hydration of the sections, the slides were treated with endogenous peroxidase in 0.3% H_2_O_2_ for 30 min, followed by blocking for 2h at room temperature with 1.5% blocking serum (Santa Cruz Biotechnology, Santa Cruz, CA, USA) in phosphate-buffered saline (PBS). Subsequently, the slides were probed overnight with FGFR1 antibody (1:100) at room temperature in a moist chamber. Then, the specimens were washed three times in PBS and treated with Envision reagent (Dako), followed by color development using DAB reagent (Dako). Finally, the slides were counterstained with hematoxylin, dehydrated with ethanol, cleaned with xylene, and mounted. As a negative control, duplicate sections were immunostained without exposure to primary antibodies. To quantitate the FGFR1 protein expression, the mean percentage of positive tumor cells was determined in at least five random fields in each section at 400× magnification. The intensity of the FGFR1 immunoreaction was scored as follows: 1+, weak; 2+, moderate; and 3+, intense. The samples with >10% positive tumor cells and the staining at 1+, 2+, and 3+ levels were considered as FGFR1 IHC-positive.

### *FGFR1* amplification by FISH

FISH was performed on formalin-fixed paraffin-embedded tumor tissues using *ZytoLight* FGFR1/CEN 8 Dual Color Probe (ZytoVision; GeneDiagnostic Inc., Hangzhou, China) according to the manufacturer's instructions.

The SPEC FGFR1/CEN 8 dual color probe is a mixture of orange fluorochrome-labeled CEN 8 probe specific for the alpha satellite centromeric region of chromosome 8 (D8Z2) and green fluorochrome-labeled SPEC FGFR1 probe specific for the *FGFR1* gene localized at 8p11.23-p11.22.

About 4-µm-thick formalin-fixed paraffin-embedded sections were deparaffinized, treated with warmed heat pretreatment citric buffer at 98 °C and digested in pepsin solution. The probe (10 μL) was added to each slide. Target DNA and probes were co-denatured at 75 °C for 10 min and incubated at 37 °C overnight in a humidified hybridization chamber, followed by three washes with 1× Wash Buffer A at 37 °C for 5 min each. Finally, the slides were air-dried and counterstained with DAPI/antifade solution. The signals for each locus-specific FISH probe were assessed under an Olympus BX51 microscope (Olympus Corporation, Tokyo, Japan) and evaluated by two pathologists. The site of interest in the FISH analysis was correlated with the histomorphology observed by hematoxylin-eosin staining.

Twenty contiguous tumor cell nuclei from three hotspots or random areas, resulting in a total of 60 nuclei, were individually evaluated at 100× by counting green FGFR1 and orange centromere 8 (CEN8) signals.

Cases were considered as FGFR1-positive (“amplified”) under one of the following conditions 15: a high-level amplification: (1) the FGFR1/CEN8 ratio is ≥2.0; (2) the average number of FGFR1 signals per tumor cell nucleus is ≥6; (3) the percentage of tumor cells containing ≥15 FGFR1 signals or large clusters is ≥10%; A low-level amplification: the percentage of tumor cells containing ≥5 FGFR1 signals is ≥50%.

### FGFR1 mutation detection by PCR

**Figure 1:**
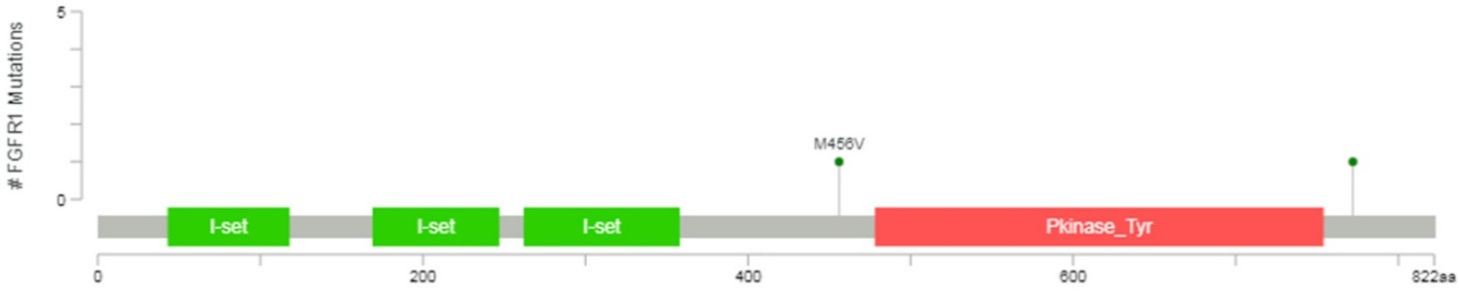
Diagrammatic representation of FGFR1 mutation from www.cbioportal.org.

The datum showed that the protein kinase regions of FGFR1 were between 478 and 754 amino acids, representing with the orange area in the **Figure 1**, so the mutational analyses of FGFR1 (exons 10-16) were performed in 33 SCLC postoperative tissue specimens using PCR amplification and Sanger sequencing in this study. The primers used for sequencing and methods were referenced by previous study (Supplementary Digital Content 1, http://links.lww.com/JTO/A534) 16.

### FGFR1 mRNA analysis by RT-PCR

After standard tissue sample deparaffinization using xylene and alcohols, RNA was isolated by EASYspin FFPE RNA isolation kit (Beijing Aidlab Biotechnologies Co., Ltd. Beijing, China). cDNA was synthesized using M-MLV retrotranscriptase enzyme. Template cDNA was added to 2×SYBR premix (Bioer Technology Co., Ltd. Hangzhou, China) in a 20 μl reaction with specific primers for FGFR1 gene. Quantification of gene expression was performed using the Mx3000P Thermal Cycler (Agilent StrataGene). Cycling conditions were 95℃ for 10 min and followed by 45 cycles at 95℃ for 15 s and 60℃ for 30 s. Relative gene expression quantification was calculated according to the comparative Ct method using GAPDH as an endogenous control. The primer sequences used for FGFR1-F: 5'-CGCCAGGACCCGAACAG-3' and FGFR1-R: 5'- CAGTGAGCTCGATCCTCCTTT-3'. *GAPDH*-F: 5'-GAAGGTGAAGGTCGGAGTC-3', GAPDH-R: 5'- GAAGATGGTGATGGGATTTC-3'.

### Follow-up

The follow-up deadline was January 1, 2020. Consequently, 14 patients were alive, 18 patients were dead, and one patient was lost to follow-up. The survival time was calculated from the date of pathological diagnosis, or last available follow-up, while relapse-free survival (RFS) until the first documentation of recurrence.

### Statistical analysis

Statistical analysis was performed using SPSS 15.0 statistical software (SPSS Inc., Chicago, IL, USA). The correlation between FGFR1 expression and amplification and clinicopathological characteristics (including age, gender, smoking, tumor size, lymph node metastasis, and TNM stage) was evaluated by Pearson's chi-square test. The survival curves were plotted by the Kaplan-Meier method, and the differences in the survival rate were assessed using the log-rank test. Univariate and multivariate analyses of prognostic factors were performed using the Cox proportional hazards model. A two-sided p-value<0.05 was considered as statistically significant.

## Results

### Prevalence of FGFR1 Protein Expression

**Figure 2:**
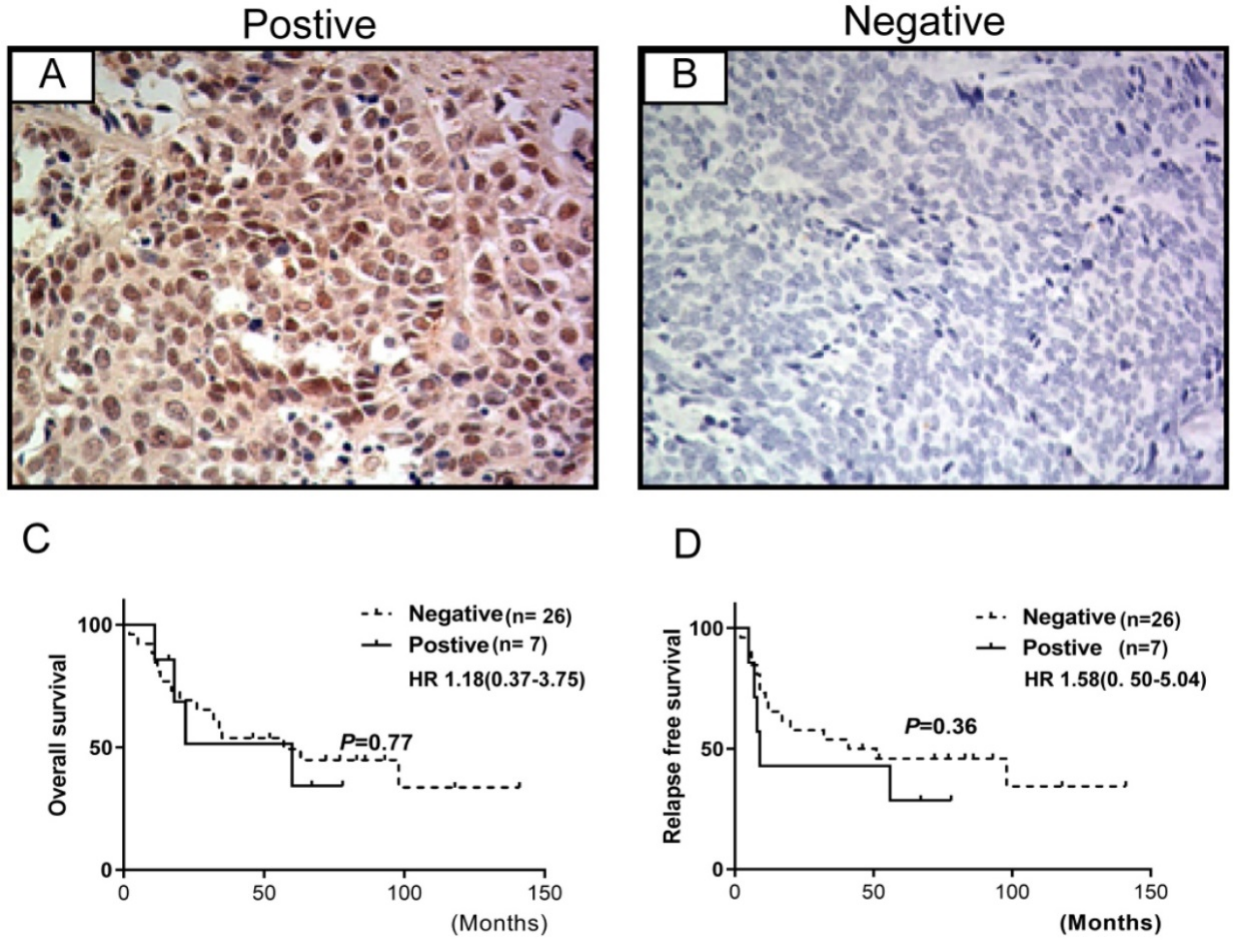
** FGFR1 protein expression by immunohistochemistry in SCLC and their correlations with prognosis. (A**) shows FGFR1 protein positive expression; (**B**) shows FGFR1 protein negative expression. Kaplan-Meier Survival analysis of FGFR1 protein-positive vs. FGFR1 protein-negative (**C and D**).

The expression of FGFR1 was evaluated in 33 specimens of SCLC cases and was observed in the cytoplasm and/or the membrane (**Figure 2**). These observations were consistent with those observed previously 8. 7/33 (21.2%) specimens were positive for FGFR1 protein expression that was not associated with prognosis. However, no significant association was observed between FGFR1 protein expression and clinical characteristics (age, gender, smoking status, lymph node metastasis, tumor stage, or brain metastasis) (**Table 2**) or relapse-free survival (RFS) and overall survival (OS) (all log-rank *p*-values >0.05) (**Figure 2**).

### Prevalence of *FGFR1* amplification by FISH

**Figure 3:**
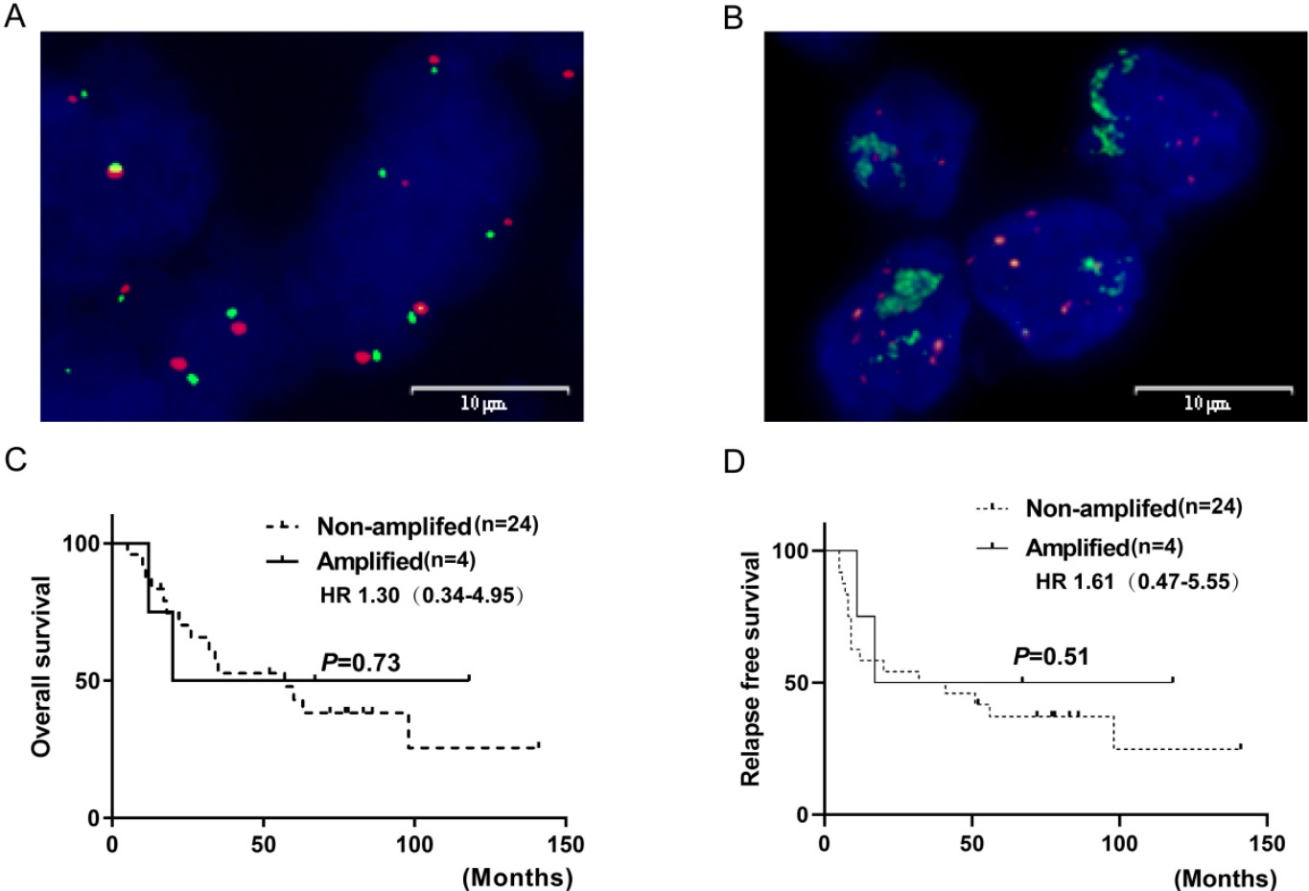
***FGFR1* amplification by fluorescence *in situ* hybridization in SCLC and their correlations with prognosis.** (**A**) shows* FGFR1* amplification (**B**) shows* FGFR1* non-amplification. Kaplan-Meier Survival analysis of* FGFR1* amplified vs. non-amplified tumors (**C and D**).

*FGFR1* gene copy number (GCN) was evaluated by FISH in 28/33 SCLC specimens. Those that could not be evaluated either lacked qualified tissue due to the prolonged storage or inadequate for a tested biomarker because of a few viable tumor cells. FGFR1 amplification, defined as at least four FGFR1 signals per nucleus or FGFR1/CEN8 ratio at 2.0, was identified in 4/28 cases (14.3%). Three amplified cases were positive for both high *FGFR1* GCN and high FGFR1/CEN8 ratio. In the amplified cases, the mean FGFR1signal per nucleus was 11.4 (range, 4.95-21.50) and the mean FGFR1/CEN8 ratio was 2.86 (range, 2.00-4.55). Thus, the *FGFR1* amplification might be caused by GCN gain on the chromosome than chromosome polysomy, as none of the positive specimens exhibited more than three CEN8 signals per nucleus. Furthermore, no significant association was observed between *FGFR1* amplification and clinical characteristics (age, gender, smoking status, lymph node metastasis, tumor stage, or brain metastasis) (**Table 2**). *FGFR1* amplification was not associated with RFS and OS (all log-rank p-values >0.05) (**Figure 3**).

### Prevalence of FGFR1 mRNA by RT-PCR

**Figure 4:**
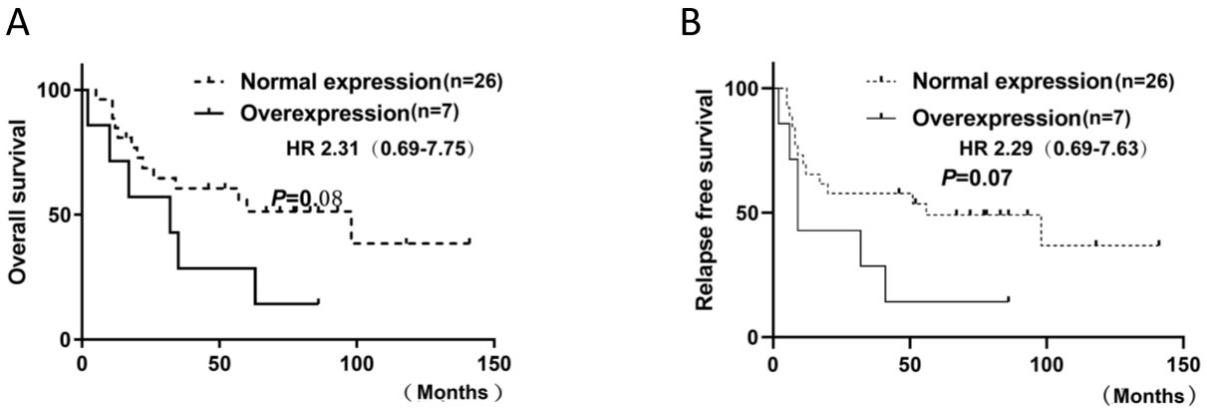
Kaplan-Meier Survival analysis of FGFR1 mRNA. (**A and B**).

FGFR1 mRNA was evaluated by RT-PCR in 33 SCLC specimens. △CT values were used to indicate mRNA expression levels. Evaluation of 33 SCLC specimens revealed 45.5% (15 of 33) with a positive score defined as at least 3; 21.2% (7 of 33) had a score of 3.5; 6% (2 of 33) had a score of 4. As there is no standard definition for positivity of mRNA, we defined at least 3.5 as the cutoff for mRNA positivity based on the presence of mRNA signal dot clusters in cases with a score of 3.5 or higher. No significant association was observed between FGFR1 mRNA expression and clinical characteristics or prognosis. However, there was a distinct trend for mRNA level and poor prognosis, including RFS (*p*=0.07) and OS (*p*=0.08), but they did not reach statistical significance (Figure. 4). A significant association between FGFR1 mRNA and age was observed with the Fisher's exact test (*p* = 0.03) (**Table 2**).

### Status of* FGFR1* mutations

Exons 10-16 of the *FGFR1* gene were sequenced by PCR amplification and Sanger sequencing. No mutation was detected in the 33 SCLC postoperative tissue specimens.

## Discussion

SCLC is a lethal disease lacking effective therapeutic options. Scientific studies about SCLC molecular profiles are hampered by a lack of tissue availability. In recent years, the systemic efforts have revealed specific therapeutic targets although limited to small samples or liquid biopsy. Recent genomic analysis of a set of SCLC tissue samples and plasma cell-free DNA revealed focal *FGFR1* amplification among other molecular aberrations 5, 17, 18. In our study, not only were all specimens obtained from surgery, that combined small-cell lung cancer can be excluded and molecular profiles were more accurately reflected than biopsy specimens, but also follow-up was more than 5 years. Furthermore, It is worth mentioning that, for the first time, we used postoperative specimens to comprehensively analyze FGFR1 alterations, including mRNA level, protein level, mutations, and gene amplification.

FGFR-mediated signaling is a promising target for cancer therapy 19, which initiates signal transduction cascade to regulate angiogenesis, cell proliferation, migration and survival. The FGFR tyrosine kinase family comprises of four kinases: FGFR1, FGFR2, FGFR3, and FGFR4 20,21. *FGFR1* is localized on the short arm of chromosome 8 (8p12). *FGFR1* alterations have been described in several tumor types, including lung cancer, breast cancer, head and neck squamous cell cancers, and esophageal cancers 22-25. Especially, Recent processes in molecular biology have confirmed that* FGFR1* amplification is detected in approximately 20% of lung squamous cell carcinoma (SqCC) 19 and is associated with the mechanism of acquired resistance to EGFR-TKIs 26,27. Also, it is observed in neuroendocrine tumors consisting of carcinoids of the lung 28. The activating alterations in *FGFR1* appear as amplification as assessed by large-scale NGS in SCLC 5,18,29. FGFR1 theoretically may be a promising potential target in SCLC, yet it is curious that FGFR1-amplified lung cancer patients experienced limited benefit from FGFR inhibition. Therefore, a better understanding of FGFR1 alterations is necessary in order to develop more effective therapeutic options. Compared with other similar studies, we had a more comprehensive evaluation of FGFR1 status using postoperative tissue samples, including amplification, mutation, and protein and mRNA level changes.

The frequency of FGFR1 protein expression in the SCLC cohort (21.2%) was higher than that in the study by Zhang et al. (7.2%) 8, and lower than that in the study by Yang et al. (43.7%) 30. This difference might be attributed to the difference in the specimen type, antibodies used (Origene *vs.*Abcam), scoring protocol, cutoffs for positivity, or cohort characteristics. Thus, developing standardized methods for IHC-based evaluation of FGFR1 protein expression is essential. Strikingly, the stability of the FGFR1 protein was putatively affected in the study by Zhang et al. because specimens stored for a prolonged period might be the reason for the low detection rate. The comparison of FGFR1 protein expression with the stage was inconclusive due to the small number of positive samples (*n* = 7). The study by Yang et al. demonstrated that the FGFR1protein expression in SCLC might be associated with late stage 30.

The frequency of *FGFR1* amplification in the current study (14.3%, n=28) was slightly higher than that previously (1.9-7.8%) 8,16,31,32. The majority of the studies used FISH to detect the *FGFR1* amplification; the frequency of *FGFR1* amplification in prior SCLC studies was 5.6-7% by FISH 16,32 or 7.8% by silver *in situ* hybridization (SISH) 8 in Western population with SCLC. However, the frequency of *FGFR1* amplification detected by FISH is lower (1.9%) in Korean patients. The small biopsy specimens rather than ethnic differences might be attributed to the failure in identifying the small number of cases of *FGFR1* amplification. These differences in *FGFR1* amplification prevalence might be due to several factors, including disease stage, specimen size, cutoffs for positivity, or detection method.

The frequency of FGFR1 mRNA positive expression in this current SCLC study (21.2%, n=33) was similar to that in prior studies of SCLC (19.7%, n = 76) 8. Our study used PCR, but the latter study used *situ* hybridization (ISH) to detect the mRNA. There are no other similar studies about mRNA level.

Any mutations of FGFR1 exons did not found in our study. So far, only two duplicate FGFR1 M456V missense mutations have been detected in tissue samples from SCLC patients 5,17, with frequency ranging from 0.9% to 3.4%, but whether there are biological roles remain unclear and need to be further verified. It's worth noting that this point mutation is not in the protein kinase regions.

However, whether *FGFR1* alterations affect the survival remains controversial. Yang et al. demonstrated that high FGFR1 protein expression correlated with poor OS and RFS 30. Conversely, neither study found any statistical significance between clinical outcome and FGFR1 protein expression and amplification 31,32. On the other hand, there are tendencies in certain cohorts of the two studies, one by Schultheis et al. found that patients with limited-stage disease and no amplification of FGFR1 exhibited satisfactory OS in their patient cohort 32, the other by Park et al. showed that among the patients with extensive-stage disease, *FGFR1* amplification was associated with short disease-free survival post-first-line chemotherapy with etoposide plus cisplatin or carboplatin 31. The results of our cohort support the latter, but not the former. However, there is trend between mRNA expression and poor OS and RFS, rather than protein expression or amplification. Of the seven mRNA positive expression cases, six patients died, but one patient with stage IA was survived. Thus, we think mRNA expression as potential marker for predicting prognosis of surgical resection of SCLCs will be better than IHC and FISH.

Furthermore, the sufficiently robust predictive biomarkers for FGFR-TKIs sensitivity are yet to be identified. Previous clinic trials enrolled based on elevated FGFR1 amplification 9. However, FGFR1 amplification seems insufficient to predict prognosis and the response to anti-FGFR therapy. Until Wynes et al. demonstrated that expression *FGFR1* mRNA is a biologically relevant marker of FGFR1 TKI sensitivity in lung cancers of all histologies, including squamous, adenocarcinoma, and SCLC among 58 cell lines *in vitro* 10, it was grew in awareness that FGFR1 amplification may not be the right biomarker to predict response 33. Eventually, just published result from a phase 1 clinical trial suggested that rogaratinib, a novel kinase inbibitor of FGFR1-4, resulted in an encouraging objective response (15%) 11, if screened by FGFR mRNA overexpressing cancers, which compares favorably with the objective response observed with other selective pan-FGFR inhibitors in early clinical trials, such as AZD4547 (8%), infigratinib (BGJ398) (5%) 9, and Erdafitinib (JNJ-42756493) (11%) 34. Selection by mRNA expression could be a useful additional biomarker not only to mildly improve efficacy but also enlarge a broader cohort who might benefit from FGFR-TKIs. As mentioned initially, results from ongoing early clinical trials would predict the optimal biomarker. Our results would set the stage for clinical trials in Chinese patients.

Nevertheless, the recent clinical trials and findings showed limited efficacy of FGFR-targeted therapy in multiple malignant tumors, suggesting that combination therapy may be essential to improve the EGFR1-amplified patient outcomes 35-37. Golfmann et al. postulated synergistic treatment effects in FGFR1/VEGFR1-positive breast cancer patients by dual targeting of FGFR and VEGFR 36. Weeden et al. identified that triple BCL-XL, MCL-1, and FGFR inhibition resulted in regression of tumor volume and prolonged in vivo survival using patient-derived xenografts, thereby demonstrating the ability of BCL-XL and MCL-1 proteins to compensate for each other in lung SqCC 37. In addition, the validated findings and lung cancer TCGA data unveiled the overlap of FGFR1 mRNA positivity with mutations in *KRAS* and *PIK3CA* genes 10. A refreshing study demonstrated that dual EGFR and FGFR blockade may be a promising clinical strategy for both preventing and overcoming acquired EGFR-TKI resistance induced by epithelial-to-mesenchymal transition (EMT) and provide motivation for the clinical study of combined EGFR and FGFR inhibition in EGFR-mutated NSCLCs^27^, ^38^. These findings indicated a partial overlap of FGFR1 dependency with distinct oncogene drivers. Therefore, comprehensive genomic profiling is required to establish robust prognostic markers in this tumor type with an in-depth understanding of the underlying mechanisms.

Several limitations of this study should be acknowledged. First of all, the sample size was too small and this was retrospective study, suggesting the findings should be interpreted with caution. To make up for the lack of the adequate sample size, we intended to conduct a meta-analysis or use various public database of cancer research, such as The Cancer Genome Atlas (TCGA), to validate these findings. Unfortunately, the meta-analysis failed to be performed due to lack of reported studies in this same/similar topic. We actually can't even find the subtype of SCLC in TCGA. And in fact, post-operative tissue samples from small cell lung cancer are rare. Up to now, there is limited availability of tissue for molecular studies due to difficulties in obtaining sufficient tumor samples in SCLC, which is the dilemma of transformation research of SCLC. Although the sample is small, we hope our study can help perform and report research on available SCLC tissue to advance the identification of novel targets in this disease.

## Figures and Tables

**Table 1:** Clinical characteristics of 28 patients with SCLC

Characteristics	Cases (n)
**Sex**	
Male	22
Female	6
**Age**	
<60 years	15
≥60 years	13
**Smokers**	
Non‑smokers	6
Light smoker (≤10 pack-years)	2
Moderate smoker (10-20 pack-years)	2
Heavy smoker smokers (≥20 pack-years)	18
**Stage**	
IA	9
IB	1
IIA	0
IIB	5
IIIA	12
IIIB	1

**Table 2:** Clinicopathological data of the patients with SCLC and FGFR1 status

Factors	FGFR1 DNA			FGFR1 mRNA (≥3.5)			FGFR1 Protein		
	Amplified	Non-amplified	p-value	Over-expression	Normal	p-value	Positive	Negative	p-value
**Gender**									
Male	4	18		5	22		7	20	
Female	0	6	0.55	2	4	0.58	0	6	0.30
**Age**									
<60	2	13		1	18		5	14	
≥60	2	11	1.00	6	8	**0.03**	2	12	0.67
**Smoking**									
Never and light smokers	0	8		2	6		0	8	
Moderate and heavy smokers	4	16	0.30	5	20	1.00	7	18	0.15
**Stage**									
I	1	11		2	12		5	9	
II-III	3	13	0.61	5	14	0.67	2	17	0.11
**Lymph node metastasis**									
N0	0	4		3	10		4	9	
N+	4	14	0.27	4	16	1.00	3	17	0.39
**Brain metastasis**									
No	2	20		7	20		4	23	
Yes	2	4	0.19	0	6	0.30	3	3	0.09

Fisher exactly test.

## Abbreviations

FGFR1: Fibroblast growth factor receptor 1
SCLC: small cell lung cancer
mRNA: messenger RNA
IHC: immunohistochemistry
FISH: fluorescence *in situ* hybridization
RT-PCR: reverse transcription-polymerase chain reaction
NGS: next-generation sequencing
TKI: tyrosine kinase inhibitor
RFS: recurrence-free survival
OS: overall survival
SqCC: lung squamous cell carcinoma
SISH: silver *in situ* hybridization
ISH: *in situ* hybridization
EMT: epithelial-to-mesenchymal transition
